# Preterm Cesarean Delivery and Safety of Subsequent Delivery: Risk of Uterine Rupture and Other Maternal and Neonatal Outcomes—Multicenter Retrospective Cohort Study

**DOI:** 10.3390/jcm14051522

**Published:** 2025-02-24

**Authors:** Sarit Helman, Shira Fridman Lev, Amy Solnica, Orna Reichman, Rivka Farkash, Sorina Grisaru-Granovsky, Maayan Bas Lando

**Affiliations:** 1 Department of Obstetrics & Gynecology, The Eisenberg R&D Authority, Shaare Zedek Medical Center, Faculty of Medicine, Hebrew University of Jerusalem, Jerusalem 9103102, Israel; 2 Henrietta Szold School of Nursing, Faculty of Medicine, Hadassah Medical Center, Hebrew University, Jerusalem 9112001, Israel

**Keywords:** TOLAC (trial of labor after cesarean), uterine rupture (UR), preterm cesarean delivery

## Abstract

**Background/Objectives**: The safety of trial of labor after cesarean (TOLAC) following prior preterm low-segment transverse cesarean delivery (pCD) was compared to that following term low-segment transverse cesarean delivery (tCD) in terms of the rate of uterine rupture (UR) and adverse maternal and neonatal outcomes. **Methods**: A multicenter retrospective cohort study evaluated the delivery outcomes among women with a prior primary pCD and those with a primary tCD. The primary outcome was UR, defined as a full-thickness uterine wall defect. The secondary outcomes included maternal and neonatal morbidities. Chi-square, Fisher’s exact test, and Mann–Whitney tests, with the results reported as means ± SDs or medians + interquartile ranges (IQRs), were employed. **Results**: The cohort comprised 5340 women, including 186 with a prior pCD and 5154 with a prior tCD. The median gestational age at pCD was 28 weeks, compared to 39 weeks for tCD. Women in the pCD group had higher rates of hypertensive disorders (20.4% vs. 2.5%; *p* < 0.001). No significant difference in UR incidence was observed at subsequent delivery (0% vs. 0.6%; *p* = 0.3). However, the pCD group had higher rates of subsequent preterm delivery (19.9% vs. 4.7%; *p* < 0.01) and vaginal birth after cesarean (VBAC) success (86.1% vs. 77.3%; *p* = 0.015). Adjusted analyses showed no significant association between pCD and composite adverse neonatal outcomes (OR = 0.796, 95% CI [0.487–1.301]; *p* = 0.363). **Conclusions**: This study underscores the safety of trial of labor after a primary preterm cesarean delivery, indicating no increased risk of uterine rupture compared to term cesarean deliveries. Care should be directed toward lowering subsequent preterm delivery and its associated risks.

## 1. Introduction

Uterine rupture (UR) is a rare, acute, and potentially catastrophic obstetric complication characterized by a complete disruption of the uterine wall integrity during labor and poses significant risks to both maternal and fetal health, often leading to life-threatening hemorrhage, maternal morbidity, and adverse perinatal outcomes that may result in long-term complications [1]. The primary risk factor for UR is trial of labor after cesarean (TOLAC). Specifically, previous studies have shown that classical uterine incisions are associated with a UR risk ranging from 9 to 12%, while, with low-segment transverse incisions, the risk is approximately 0.5% [2,3]. This notable discrepancy is attributed to the location of the incision in relation to the contractile region of the uterus and the structural integrity of the uterine scar under the stress of labor [2,3]. Additional risk factors for UR include labor induction, a short interval between deliveries, an advanced maternal age, an elevated body mass index (BMI), fetal macrosomia, and the absence of a prior vaginal delivery [4,5].

The development of the lower uterine segment typically occurs during the third trimester; hence, in cases of preterm cesarean delivery (pCD), the lower segment may exhibit inadequate development, potentially resulting in the uterine incision occurring in the contractile portion of the uterus [6]. Prior investigations have yielded conflicting findings, with earlier retrospective analyses indicating an elevated risk of UR following pCD compared with term cesarean delivery (tCD), while others have reported no discernible difference in rupture rates [6,7,8,9,10,11]. These discrepancies highlight the need for further research to clarify the safety of subsequent pregnancies following pCD.

Thus, the current study aimed to compare the rate of uterine rupture (UR) and other adverse maternal and neonatal outcomes with subsequent delivery between the following two groups of women: those attempting a trial of labor after cesarean (TOLAC) following a previous pCD and those attempting a TOLAC following a tCD. By addressing this knowledge gap, our findings may contribute to more informed clinical decision making and risk assessment for women with a history of preterm cesarean delivery.

## 2. Materials and Methods

A retrospective longitudinal follow-up study of all women with a previous cesarean delivery who returned for their subsequent delivery at one of two obstetric centers—Shaare Zedek Medical Center (SZMC) and Bikur Cholim Hospital (BCH)—between 2006 and 2022 was conducted.

### 2.1. Study Population

Helman et al. described the obstetric setting of the study population in detail [12]. In brief, the two hospitals manage approximately 22,000 deliveries annually, with ~25% being nulliparous women and ~18% being grand multiparous women (parity of ≥6), predominantly from the ultra-Orthodox Jewish community. Cesarean deliveries account for ~12%, and ~5% are vacuum-assisted deliveries. Over 95% of deliveries are funded by National Public Insurance and are primarily managed by certified nurse midwives. Obstetrics and gynecology resident physicians and board-certified obstetricians are always present around the clock. Physicians manage women who require labor induction, make decisions regarding labor augmentation, and perform operative vaginal deliveries or emergency cesarean deliveries.

According to our department protocol, TOLAC is attempted in women with a history of one prior low-segment transverse CD, provided that they give their informed consent. The induction of labor in women with a prior CD includes a double lumen cervical balloon, amniotomy, and/or the use of low-dose oxytocin (oxytocin 10 IU in 1000 mL of Ringer lactate starting at 0.5 mU/min, increased by 0.5 mU/min every 20 min until 3–4 contractions per 10 min are reached) [13]. Prostaglandins are not used in women with a prior CD. Multiparous women (parity > 5) with a previous CD are not induced or augmented during labor.

The current study excluded prior intrauterine fetal death (IUFD), multiple pregnancy in primary and subsequent deliveries, and cesarean deliveries that were not low-segment transverse cesarean section (LSTCS).

### 2.2. Data Collection

Data were retrieved from electronic medical records (EMRs) and recorded in real time at the point of care by attending caregivers during labor and delivery. The summary notes in EMRs include an updated list of pertinent diagnoses following the International Classification of Diseases (ICD), which is revised by the attending physician before discharge. EMRs mainly comprise fixed, obligatory fields that must be completed before the patient is transferred to the postpartum ward. Data were retrieved for both the primary CD and subsequent delivery and included the following: maternal age, parity, assisted reproductive technology (ART) utilization, maternal hypertension and gestational diabetes, gestational age at delivery, onset of labor (spontaneous, induction, or planned CD), mode of delivery (spontaneous vaginal birth, vacuum delivery, or CD), and birth weight of newborn. Maternal adverse outcomes were defined as postpartum hemorrhage (PPH), blood transfusion, placenta accreta, uterine rupture, and uterine dehiscence. Neonatal adverse outcomes were defined as a 5’ Apgar score of < 7, intrapartum fetal death, neonatal intensive care unit (NICU) admission, jaundice, sepsis, blood transfusion, and respiratory distress syndrome (RDS). A composite of these neonatal adverse outcomes included a 5’ Apgar score of less than 7, NICU admission for more than 3 days, jaundice, sepsis, blood transfusion, and RDS.

### 2.3. Study Outcomes

The primary outcome was uterine rupture (UR), defined as a full-thickness uterine wall defect either at the site of the previous scar or another uterine location. The secondary outcomes were maternal and neonatal adverse outcomes, including preterm delivery (<37 weeks gestation), repeat CD, peripartum hysterectomy, and peripartum repeat laparotomy for bleeding control. Adjusted multivariable logistic regression was used to examine the association between preterm CD and the composite of adverse neonatal outcomes at subsequent TOLAC.

### 2.4. Statistical Analysis

Descriptive statistics were used to summarize characteristics, with proportions for nominal variables, means ± standard deviations (SDs) for normally distributed continuous variables, and medians with interquartile ranges (IQRs) for non-normally distributed continuous variables. Categorical variables were compared using the Chi-square test or Fisher’s exact test, while continuous variables were analyzed using the unpaired Student’s *t*-test or Mann–Whitney test, as appropriate.

Univariate analysis was conducted to test the associations between various maternal demographic, obstetric, and delivery characteristics and neonatal outcomes, including the composite of adverse neonatal outcomes. A *p*-value of < 0.5 was considered to be statistically significant. A multivariable logistic regression model included significant variables in the univariate analysis. The results of these analyses are reported as adjusted odds ratios (aORs) with corresponding 95% confidence intervals (CIs).

All statistical tests were two-sided, and analyses were conducted using the SPSS software (version 29; IBM, Armonk, NY, USA).

The study was conducted in accordance with the Declaration of Helsinki, and approved by the Ethics Committees of SZMC and BCH (approval number: 0216-23-SZMC). The study approval date was 2 November 2023. As this was a historical study, a waiver of informed consent was obtained.

## 3. Results

**Figure 1:**
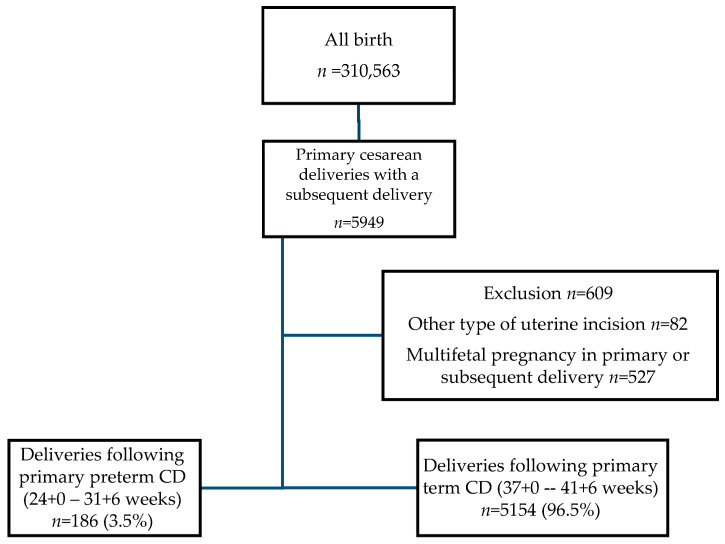
Study population.

During the study period, 5340 deliveries met the criteria. Among the subsequent deliveries, 186 (3.5%) were following a primary pCD, while 5154 (96.5%) were following a primary tCD (Figure 1).

The primary pCD group included 59 cases between 24 + 0 and 27 + 6 weeks of gestation and 127 cases between 28 + 0 and 31 + 6 weeks, while the primary tCD group included 1777 cases between 37 + 0 and 38 + 6 weeks, 2498 cases between 39 + 0 and 40 + 6 weeks, and 879 cases between 41 + 0 and 41 + 6 weeks. Among the pCD group, all cesarean deliveries were performed in an emergency setting. The indication for cesarean delivery was maternal in 45 cases due to severe preeclampsia (24%), while the remaining majority were due to fetal indications, including spontaneous preterm delivery (31%), placental abruption (15%), and fetal distress (30%). As detailed in Table 1, the pCD and tCD groups differed at the time of the primary cesarean delivery in terms of maternal age, parity, pregnancy hypertensive disorders, and, as can be expected, gestational age at delivery and neonatal birthweight.

At the subsequent delivery, the mean gestational age was lower in the pCD group compared to the tCD group (38 vs. 39 weeks, *p* < 0.001). However, the median gestational age was 39 weeks in both groups. The rate of preterm delivery (<37 weeks) was significantly higher in the pCD group compared to the tCD group (19.9% vs. 4.7%, *p* < 0.001). There were no statistically significant differences between the two study groups regarding the induction of labor, planned cesarean delivery (CD), or the spontaneous onset of labor, as described in Table 2.

There was no statistical difference in the rate of UR between the groups, no cases in the pCD group, and 32 cases in the tCD group (0% vs. 0.6% *p* = 0.3, respectively). The mode of delivery varied between the groups; women in the pCD group had a higher rate of spontaneous vaginal deliveries compared to the tCD group (57% vs. 44%, *p* = 0.003). The tCD group had a significantly higher rate of emergent cesarean deliveries than the pCD group (14.8% vs. 10%, *p* = 0.003). In the pCD group, there was one case of peripartum hysterectomy (0.5%) compared to four cases (0.08%) in the tCD group, all due to placenta accreta (*p* = 0.04). Additionally, the tCD group had significantly lower hypertension rates than the pCD group (*p* < 0.001). Neonatal outcomes also varied between the groups. The tCD neonates had a higher mean birth weight than the pCD group (3275 g vs. 2939 g, *p* < 0.001). Additionally, the tCD group exhibited lower rates of adverse neonatal outcomes such as 1’ and 5’ Apgar scores of < 7 and NICU admission (*p* < 0.05 for all comparisons), see Table 3.

pCD was found to be significantly related to a higher rate for the composite of adverse neonatal outcomes (including a 5’ Apgar score less than 7, NICU admission for more than 3 days, newborn jaundice requiring treatment, newborn sepsis, newborn blood transfusion, and newborn RDS) in the subsequent delivery, at 24.5% vs. 15.1%; (*p* = 0.001). An adjusted multivariable logistic regression analysis was applied for significant covariates and confounders to examine the association between pCD and the composite of adverse neonatal outcomes in the subsequent delivery. When adjusting for preterm gestational age, nulliparity, mode of delivery, and newborn weight, we found no significant association between pCD and the composite of adverse neonatal outcomes (OR = 0.794, 95%CI [0.486–1.298]; *p* = 0.358), see Table 4.

## 4. Discussion

### 4.1. Principal Findings

Our study found that women who had a primary pCD did not have an increased risk of uterine rupture or other maternal adverse outcomes in subsequent deliveries compared to those with a prior primary tCD. Due to the rarity of UR, and since there were no cases of uterine rupture in the previous preterm cesarean group and that this outcome occurred in only 0.6% of the previous term cesarean group, the study was not powered for a conclusive significance analysis regarding this outcome. However, as anticipated, a higher incidence of repeat preterm delivery was noted in women with previous preterm cesarean deliveries.

### 4.2. Results in the Context of What Is Known

The National Institute of Health recognizes trial of labor as a reasonable option for women with prior cesarean deliveries, despite a lack of randomized trials comparing maternal and neonatal outcomes between TOLAC and repeat CDs [14]. However, based on earlier observational trials, TOLAC increases the risk of maternal and perinatal adverse outcomes, especially for women that have undergone unsuccessful TOLAC and repeat emergency cesarean. In previous studies, women with a primary CD were at an increased risk for uterine rupture in subsequent delivery. The hallmark study by Landon et al. [15] compared women who underwent a trial of labor and women who had an elective repeat CD without labor. In this study, symptomatic UR occurred in 0.7 percent of women, and the absolute risk for hypoxic–ischemic encephalopathy was 0.46 per 1000 women at term undergoing a trial of labor. The rate of endometritis was higher in women undergoing TOLAC, as was the rate of blood transfusion.

The American College of Obstetrics and Gynecology (ACOG) states that most maternal morbidity related to TOLAC occurs when repeat cesarean delivery becomes necessary [4]. Thus, VBAC is associated with fewer complications than elective repeat cesarean delivery, whereas an unsuccessful TOLAC is associated with more complications. VBAC is also associated with a decreased risk of complications in future pregnancies and a decrease in the overall cesarean delivery rate at the population level [16].

Information regarding the safety of TOLAC after pCD is controversial (Table 5). Sciscione et al. [9] compared uterine rupture after term > 37 weeks (*n* = 35,528) and preterm < 37 weeks (*n* = 5839) CD in 19 centers of the Maternal-Fetal Medicine Units Network and found a higher risk for UR at 0.79% vs. 0.46% (*p* = 0.001) with OR= 1.62 95% CI [1.01–2.50]). However, the study groups included women with multiple CDs (24%) and women with T and J extensions into the uterine contractile area. Harper et al. [8] conducted a study at 17 tertiary centers in the United States, comparing two groups of women with one LSTCS, those who delivered before 34 weeks and those who delivered after 34 weeks, both attempting TOLAC. Harper et al. [8] found in their study that the risk of uterine rupture (aOR, 1.5; 95% CI, 0.7–3.5; *p* = 0.32) and composite morbidity (aOR, 0.9; 95% CI, 0.5–1.8; *p* = 0.81) were similar between the two groups. Contrary to these studies, Lannon et al. [11] compared 456 subsequent deliveries after periviable CD between 20 and 27 weeks and 10,505 term CDs and found a higher risk of UR at 1.8% vs. 0.4% (*p* < 0.001, respectively, with OR = 4.7 95% CI [2.3–10.6]) in those with a periviable CD. This study included all deliveries, including elective CD, and there was a lack of obstetric data regarding hysterotomies at less than 24 weeks. Mantel et al. [6] used the Swedish Medical Birth Register and identified 9300 women with one previous pCD and 57,168 women with one previous tCD. The uterine rupture rate at subsequent delivery among women with a pCD was 102 (1.1%) compared with 759 women (1.4%) with tCD. This corresponded to a decreased risk of UR for women with pCD (OR, 0.79; 95% CI, [0.64–0.97]). However, the significance of this was annulled by multivariate analysis.

### 4.3. Mechanisms and Clinical Interpretation

The higher rate of spontaneous preterm labor observed in women with a prior pCD can be explained, at least in part, by the nature of their initial delivery. Given that all preterm cesarean sections in our cohort were performed in an emergency setting, it is likely that the underlying maternal or fetal complications leading to the initial preterm birth also predisposed these women to an increased risk of recurrent preterm labor. This is consistent with prior studies demonstrating that spontaneous preterm birth is a strong predictor of recurrence [17]. Additionally, emerging evidence suggests that emergency cesarean delivery may have long-term consequences on uterine remodeling and inflammation, which could further contribute to an increased risk of spontaneous preterm birth in subsequent pregnancies [18].

Repeated cesarean deliveries (CDs) are associated with cumulative maternal and neonatal risks that increase with each successive surgery. Studies indicate that women undergoing multiple CDs are at a higher risk for complications such as abnormal placentation, including placenta previa and placenta accreta spectrum disorders, which can lead to significant maternal morbidity and mortality [19,20]. Additionally, multiple CDs are linked to an increased risk for adhesions, surgical complications, and longer operative times [19]. Uterine rupture risk rises with the number of prior cesareans, particularly in those attempting TOLAC after multiple previous CDs [16]. Moreover, women undergoing repeated CDs have a heightened likelihood of requiring hysterectomy due to severe hemorrhage [21]. Neonatal risks also increase with each subsequent CD, including respiratory distress and preterm birth [22]. Given these risks, allowing TOLAC for women with a prior CD and reducing the overall cesarean delivery rate remain key public health objectives.

### 4.4. Clinical Implications

Our findings indicate that TOLAC may be safely considered for women with a prior pCD, potentially without an increased risk of uterine rupture, as observed by some previous studies. This study’s results can assist the clinical decision-making process and support the development of more tailored management strategies for these women.

### 4.5. Research Implications

Despite our findings, significant questions remain regarding the management of TOLAC after pCD. Future research should aim to focus on the long-term maternal and neonatal outcomes of TOLAC after pCD, investigating the role of gestational age at the time of primary CD in predicting TOLAC success and safety, conducting large-scale, multicenter prospective studies with diverse populations to establish clearer clinical guidelines, and assessing the role of individualized risk stratification tools for guiding clinical decisions on TOLAC after pCD.

### 4.6. Strengths and Limitation

The strengths of this study include its large sample size and the use of comprehensive, real-time EMRs, which enhance the reliability and generalizability of the findings. The multicenter design adds to the robustness of the results. It is important to note that, in all cases where TOLAC was permitted, we ensured that the incision type was confirmed to be LSTCS. It is possible that not all studies examining the rupture rates in deliveries following CD in earlier gestational weeks verified the primary CD’s incision type. However, the study’s retrospective nature introduces potential selection bias and limits the ability to establish causality. Additionally, we do not have data regarding the BMI of the study population, as this information was not routinely collected during these years. The study had a relatively small sample size to investigate the primary outcome. Despite these limitations, the study provides significant insights into the safety of TOLAC after primary pCD, contributing valuable evidence to the existing body of literature.

## 5. Conclusions

This multicenter retrospective cohort study underscores the safety of trial of labor after primary preterm cesarean delivery, indicating no increased rate of uterine rupture compared to term cesarean deliveries. The findings highlight the need for further research to develop clearer clinical guidelines and management strategies for TOLAC in this population. Such efforts aim to optimize perinatal outcomes while reducing the incidence of unnecessary repeat cesarean deliveries.

## Figures and Tables

**Table 1:** Characteristics of the primary cesarean section.

Delivery Characteristics	pCD *n* = 186	tCD *n* = 5154	*p* Value
Gestational age, median (range)	29 (27–30)	39 (38–40)	<0.001 ^@^
Nulliparity *n* (%)	97 (52.2)	3107 (60.3)	0.026 ^^^
Maternal age, years mean ± SD	28.9 ± 5.5	30.6 ± 5.6	<0.001 ^#^
Advanced maternal age (>35 years) *n* (%)	26 (14%)	1021 (19.8%)	0.049 ^^^
Pregnancy Comorbidities *n* (%) *			
GDM	4 (2.2%)	347 (6.7%)	0.100 ^^^
Hypertension	47 (25.3%)	238 (4.6%)	<0.001^^^
ART	12 (6.5%)	423 (8.2%)	0.400 ^^^
Neonatal birthweight, grams mean ±SD	1151 ± 385	3236 ±538	<0.001 ^@^
1’ Apgar score < 7 *n* (%)	119 (64%)	594 (11.5%)	<0.001 ^^^
5’ Apgar score < 7 *n* (%)	74 (39.8%)	129 (2.5%)	<0.001 ^^^
NICU admission *n* (%)	180 (96.8%)	420 (8.1%)	<0.001 ^^^

GDM = gestational diabetes mellitus, ART = assisted reproductive technologies, and NICU = neonatal intensive care unit. * the reference value is the absence of co-morbidities, Mann–Whitney ^@^ student *t*-test ^#^ chi-square ^^^.

**Table 2:** Characteristics of the subsequent delivery.

	History of pCD *n* = 186	History of tCD *n* = 5154	*p* Value
Gestational age, median (range)	39 (37–40)	39 (38–40)	<0.010 ^@^
Preterm delivery < 37 weeks *n* (%)	37 (19.9%)	240 (4.7%)	<0.001 ^^^
Onset of labor *n* (%)			
Induction of labor	11 (5.9%)	396 (7.7%)	0.230 ^^^
Planned CD	49 (26.3%)	1676 (32.5%)	0.050 ^^^
Spontaneous onset of labor	137 (73.7%)	3478 (67.5%)	reference
Comorbidities *n* (%) *			
GDM	10 (5.4%)	382 (7.4%)	0.296 ^^^
Hypertension	38 (20.4%)	130 (2.5%)	<0.001 ^^^
ART *n* (%)	10 (5.4%)	316 (6.1%)	0.673 ^^^

GDM = gestational diabetes mellitus and ART = assisted reproductive technologies. * the reference value is the absence of co-morbidities, Mann–Whitney ^@^ chi-square ^^^.

**Table 3:** Maternal and neonatal outcomes of the subsequent delivery.

	History of pCD *n* = 186	History of tCD *n* = 5154	*p* Value
Uterine Rupture *n* (%)	0 (0%)	32 (0.6%)	0.321 ^^^
Mode of delivery *n* (%)			
Spontaneous vaginal	106 (57%)	2290 (44%)	reference
Instrumental	13 (7%)	424 (8.2%)	0.170 ^^^
Emergent CD	18 (10%)	764 (14.8%)	0.080 ^^^
Planned CD	49 (26%)	1676 (33%)	0.090 ^^^
PPH *n* (%)	1 (0.5%)	47 (0.9%)	0.595 ^^^
Blood transfusion *n* (%)	1 (0.5%)	19 (0.4%)	0.711 ^^^
Placenta accreta spectrum *n* (%)	1 (0.5%)	6 (0.11%)	0.119 ^^^
Peripartum hysterectomy *n* (%)	1 (0.5%)	4 (0.08%)	0.044 ^^^
Uterine dehiscence *n* (%)	1 (0.5%)	82 (1.6%)	0.254 ^^^
IUFD *n* (%)	1 (0.5%)	24 (0.5%)	0.972 ^^^
Puerperal fever *n* (%)	1 (0.6%)	25 (0.6%)	0.631 ^^^
Birth weight (mean ± SD)	2939 ± 691	3275 ± 526	<0.001 ^@^
1’ Apgar score < 7 *n* (%)	21 (11.3%)	265 (5.1%)	<0.001 ^^^
5’ Apgar score < 7 *n* (%)	8 (4.3%)	96 (1.9%)	0.018 ^^^
NICU admission *n* (%)	30 (16.1%)	291 (5.6%)	<0.001 ^^^
Jaundice *n* (%)	19 (10.9%)	327 (6.7%)	0.031 ^^^
Sepsis *n* (%)	4 (2.3%)	45 (0.92%)	0.087 ^^^
RDS *n* (%)	15 (8.6%)	192 (3.2%)	0.002 ^^^
Neonatal hypoglycemia *n* (%)	11 (6.3%)	348 (7.1)	0.678 ^^^
Neonatal blood transfusion *n* (%)	3 (1.6%)	12 (0.24%)	0.014 ^^^
Composite of neonatal adverse outcomes *n* (%)	40 (24.5%)	634 (15.1%)	0.001 ^^^

PPH = postpartum hemorrhage, IUFD = intrauterine fetal death, NICU = neonatal intensive care unit, and RDS = respiratory distress syndrome. Mann–Whitney ^@^ chi-square ^^^.

**Table 4:** Multivariable model for composite neonatal adverse outcomes *.

Variable	Crude OR (95% CI)	Adjusted OR (95% CI)	*p*-Value
Study Group history of pCD (Reference = history of tCD)	1.826 (1.265–2.634)	0.794 (0.486–1.298)	0.358
Nulliparity at index delivery	0.989 (0.836–1.171)	0.946 (0.780–1.147)	0.571
Preterm Birth (<37 weeks)	20.854 (15.640–27.808)	12.684 (9.059–17.760)	<0.001
Mode of Delivery			
—Vaginal (Reference)			
—Instrumental	1.220 (0.916–1.624)	1.939 (1.397–2.691)	<0.001
—Cesarean Delivery	1.399 (1.186–1.651)	1.259 (1.032–1.536)	0.023
Macrosomia	0.809 (0.576–1.135)	1.131 (0.797–1.604)	0.491
Low Birth Weight (LBW)	8.987 (6.984–11.566)	2.700 (1.922–3.792)	<0.001

* composite variable of neonatal adverse outcomes: the presence of at least one of the following conditions: Apgar score ≤ 7 at 5 min, NICU stay > 3 days, neonatal jaundice requiring treatment, newborn sepsis, newborn blood transfusion, or neonatal RDS.

**Table 5:** Studies aimed to assess TOLAC after pCD: brief summary.

	Design	Sample Size	Primary Outcome	OR (95% CI)
Sciscione et al., 2008 [9]	Prospective observational <37 weeks	5839	Uterine rupture	1.62 (1.01–2.50)
Harper et al., 2009 [8]	Retrospective cohort <34 weeks	508	Maternal morbidity, including uterine rupture	No difference
Rochelson et al., 2009 [7]	Case-control <36 weeks	25	Uterine rupture	5.39 (2.3–12.4)
Lannon et al., 2015 [10]	Retrospective longitudinal 20–27 weeks	456	Uterine rupture	4.7 (2.3–10.6)
Mantel et al., 2021 [6]	Prospective <32 weeks, 32–36 weeks	9300	Uterine rupture	No difference

## Data Availability

The deidentified dataset will be made available upon reasonable request.

## Abbreviations

The following abbreviations are used in this manuscript:

LSTCS	Low-segment transverse cesarean section
ACOG	American College of Obstetrics and Gynecology
aOD	Adjusted Odds ratio
ART	Assisted reproductive technologies
BCH	Bikur Cholim Hospital
BMI	Body mass index
CI	Confidence interval
EMR	Electronic medical record
GDM	Gestational diabetes mellitus
ICD	International Classification of Diseases
IQR	Interquartile range
IUFD	Intrauterine fetal death
NICU	Neonatal intensive care unit
pCD	Preterm cesarean delivery
PPH	Postpartum hemorrhage
RDS	Respiratory distress syndrome
SD	Standard deviations
tCD	Term cesarean delivery
TOLAC	Trial of labor after cesarean
UR	Uterine rupture
VBAC	Vaginal birth after cesarean
